# Supplementary material for: Comparison of longitudinal trends in self-reported symptoms and COVID-19 case activity in Ontario, Canada
Source: PLoS One. 2022 Jan 11;17(1):e0262447. doi: 10.1371/journal.pone.0262447 (PMC8754059; doi:10.1371/journal.pone.0262447)
Supplement: S1 Appendix — (DOCX) [file pone.0262447.s001.docx]

**Comparison of longitudinal trends in self-reported symptoms and COVID-19 case activity in Ontario, Canada**

**Supporting Information – S1 Appendix**

**S1 Appendix**

**
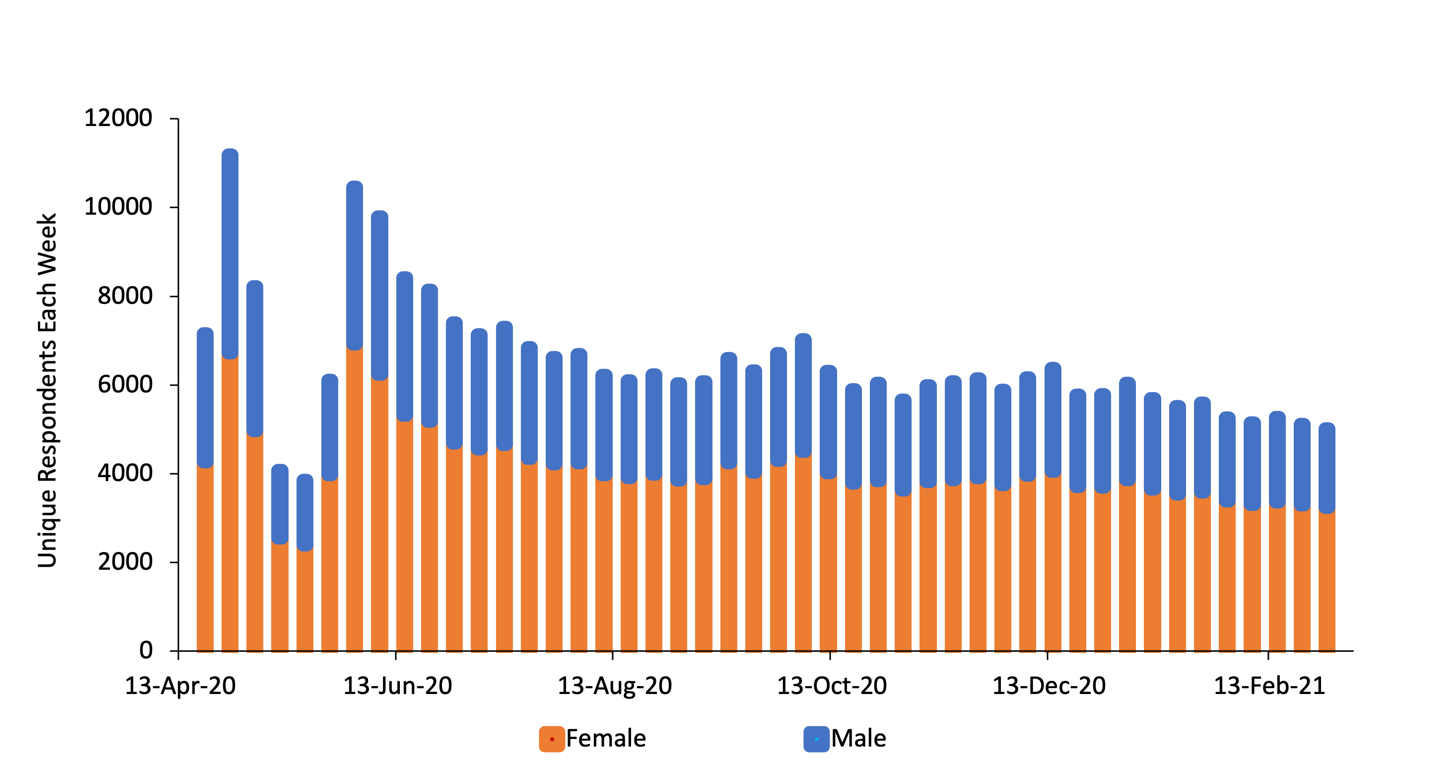
**

**S1 Figure 1:** Total number of unique respondents each week from the Outbreaks Near Me survey

**
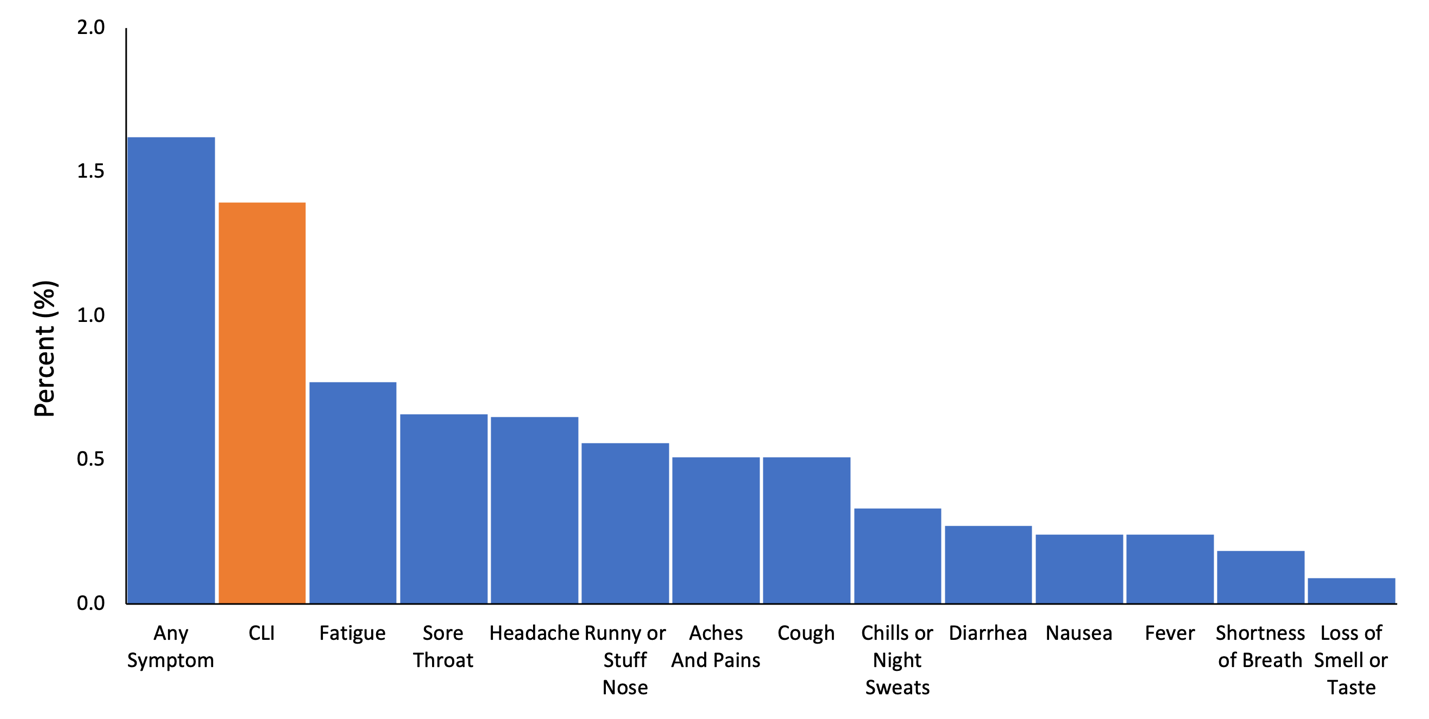
S1 Figure 2:** Percent of responses reporting each symptom from the Outbreaks Near Me survey. COVID-like Illness (CLI)

**
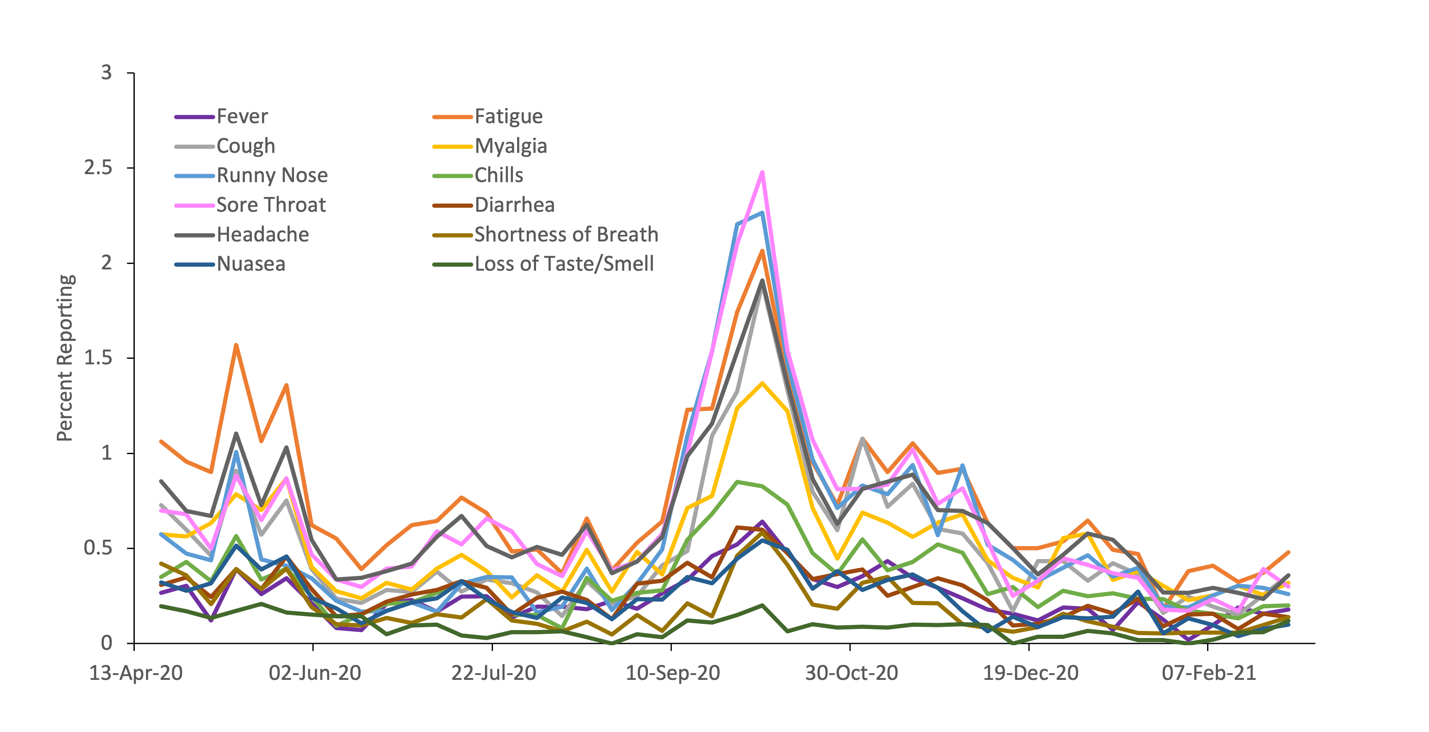
S1 Figure 3:** Percent of responses reporting each symptom over time from the Outbreaks Near Me survey

**
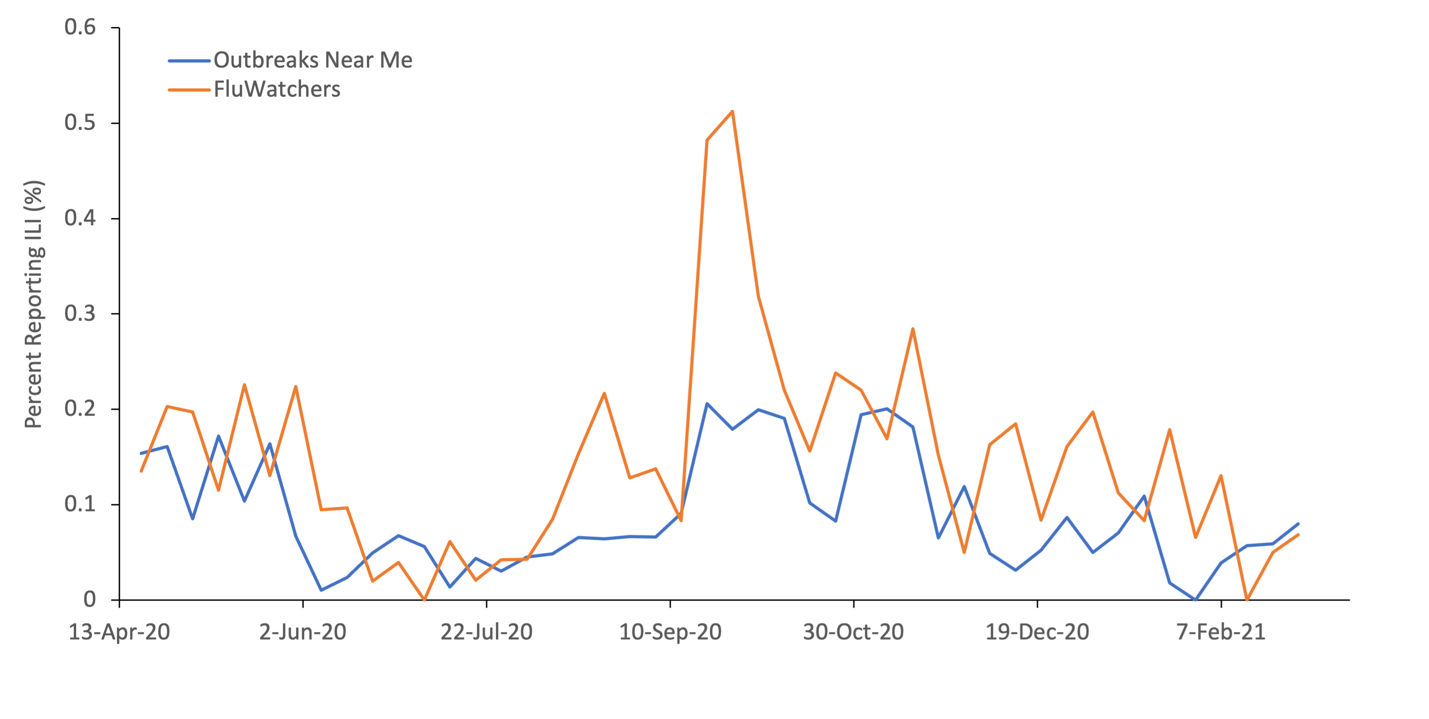
**

**S1 Figure 4:** Proportion of individuals reporting ILI (both fever and cough) from Outbreaks Near Me and FluWatchers

**S1 Figure 5:** Percent CLI and CLI_3_ vs new weekly COVID-19 cases

**S1 Figure 6:** Percent of respondents reporting direct contact with a laboratory confirmed case of COVID-19, along with those with direct contact and symptoms compared to weekly COVID-19 cases in Ontario

**S1 Table 1: Correlations between CLI and COVID-19 Parameters in Ontario**

| **Parameter (Percent CLI from ONM vs:)** | **Entire Time Period**  (Week 17 – week 9) | **Before Spike in Rhinovirus**  (Week 17 – Week 34) | **During Spike in Rhinovirus**  (Week 35 – week 2) | **After Spike in Rhinovirus**  (Week 2 – Week 9) |
| --- | --- | --- | --- | --- |
| COVID-19 cases | 0.02 | 0.65^*^ | -0.36 | -0.07 |
| One-week future COVID-19 cases | 0.06 | 0.66^*^ | -0.37 | 0.26 |
| COVID-19 test positivity | 0.09 | 0.64^*^ | -0.34 | 0 |
| COVID-19 one-week future test positivity | 0.16 | 0.64^*^ | -0.35 | -0.09 |
| COVID-19 cases by symptom onset date | 0.04 | 0.44 | -0.43 | -0.64 |
| Symptomatic COVID-19 cases | 0.01 | 0.64^*^ | -0.36 | -0.04 |
| COVID-19 Cases ≥19 years old^†^ | 0.01 | 0.67^*^ | -0.36 | -0.04 |
| CLI_2_^**^ | 0.09 | 0.74^*^ | -0.29 | 0.11 |
| CLI_3_^**^ | 0.05 | 0.65 | -0.27 | -0.11 |

^*^Spearman correlation coefficient significant at p<0.05

^†^COVID-19 cases ≥19 years of age were compared to percent CLI of those ≥19 years of age

^**^CLI_2_ and CLI_3_ was compared to weekly total COVID-19 cases
