# Supplementary material for: Comparison of longitudinal trends in self-reported symptoms and COVID-19 case activity in Ontario, Canada
Source: PLoS One. 2022 Jan 11;17(1):e0262447. doi: 10.1371/journal.pone.0262447 (PMC8754059; doi:10.1371/journal.pone.0262447)
Supplement: Attachment — Submitted filename: Response to reviewers.docx [file pone.0262447.s003.docx]

**Response to Reviewers RE:** Comparison of longitudinal trends in self-reported symptoms and COVID-19 case activity in Ontario, Canada

**Reviewer #1**

**1.) The major issue comes from definition of COVID-like illness (CLI), defined by**

**“the presence of at least two of: fever (measured or subjective), chills, ... or new taste disorder. “Though they cited these symptoms from CDC website (https://ndc.services.cdc.gov/case-definitions/coronavirus-disease-2019-2021/), the CDC has never defined these symptoms as CLI. Indeed, on the same webpage, under the section of “Case Classification > Probable”, the CDC requires a case to be probable, under the condition when no confirmatory or presumptive laboratory evidence for SARS-CoV-2 is available, to have at least “epidemiologic linkage” which is not reported in current study BUT can surely be obtained using a participatory approach, as shown in other published studies**

Thank you for this excellent point on including epidemiologic linkage which was not reported in our original submission. Indeed, this was available in the Outbreaks Near Me tool. We have added an additional analysis to our manuscript where we compare the proportion of weekly participatory surveillance respondents who reported close contact to confirmed COVID-19 case to weekly cases in Ontario. This was also combined this with symptom data such that those with both CLI and contact were compared to weekly cases. This resulted in a new CLI definition as suggested: CLI + epidemiologic linkage.

We found that there was a strong association between the proportion of those reporting close contact and cases in Ontario (ρ = 0.77). There was also a strong association between those with contact + CLI symptoms and cases in Ontario (ρ = 0.70). Close contact did track with the second wave in Ontario while symptom data alone did not. These were expected findings as self-reported close contact reflects burden of disease in an area. This finding has been added to Supplementary figure 6 with integration into the discussion and conclusion section.

**Reviewer #2**

**1.) Move Historical Trends of COVID-19 to backgrounds section**

Thank you for this recommendation. Our section on historical trends has now been moved to the background section

**2.) Missing citation for 2016 census methodology**

We apologize for this oversight. A citation has been added for the 2016 census methodologies

**3.) Strengthening discussion**

Thank you for this recommendation. We have added additional points to our discussion including further commentary on similarities in trends seen between different syndromic definitions, commentary on adding epidemiologic linkage to our syndromic definitions, and additions to the strengths section of the manuscript.

**4.) Conclusion Section**

We have added two additional conclusion points namely on our new finding of self-reported direct contact and integrating our finding on differences between participatory respondent demographic and Ontario population demographics.

**Editor comments**

**To fully assess the use of participatory surveillance using a symptom only approach, the authors may consider other combinations symptom which may be less affected by other respiratory infections (e.g. rhinoviruses or enteroviruses). Such alternative definitions have been considered in Reses et al. (BMC Public Health, 2021).**

Thank you for this suggestion. We agree that testing other combinations of symptoms are important to uncover one that may not be affected by other infections. We have reviewed Reses et al. in depth and have chosen a symptom combination with the highest specificity for COVID-19 that was also available with the symptoms surveyed by ONM. This was added to our paper as CLI_3_ and consisted of taste and/or smell dysfunction, or one of the following: shortness of breath, myalgia, or fever or chills. This was listed as derived compound combination 1 in Reses et al and added to Supplement Figure 5. We found that this symptom combination also did not correlate with weekly cases and in fact also followed the Rhinovirus spike. We believe it to be a strength of our paper to now have 4 syndromic definitions. All 4 showed very similar trends over time. Further, we included a breakdown of all symptom data over time in Supplement Figure 3. Here we see all symptom components track with each other (all experiencing a rhinovirus spike) indicating that it is unlikely that a specific combination of symptoms would track only with COVID-19 in Ontario. This indicates the heavy syndromic overlap between COVID-19 and other respiratory viruses.
